# RIPC provides neuroprotection against ischemic stroke by suppressing apoptosis via the mitochondrial pathway

**DOI:** 10.1038/s41598-020-62336-w

**Published:** 2020-03-24

**Authors:** Jing Lv, Weikang Guan, Qiang You, Li Deng, Yan Zhu, Kan Guo, Xiaoqing Gao, Jiming Kong, Chaoxian Yang

**Affiliations:** 1 Department of Neurobiology, Preclinical Medicine Research Center, Southwest Medical University, Luzhou, 646000 China; 2 The First People’s Hospital of Ziyang, Ziyang, 641300 China; 3 Department of Nuclear Medicine, Affiliated Hospital of Southwest Medical University & Nuclear Medicine and Molecular Imaging Key Laboratory of Sichuan province, Luzhou, 646000 China; 4 Department of Anatomy, College of Basic Medicine, Southwest Medical University, Luzhou, 646000 China; 5 Department of Human Anatomy and Cell Science, Rady Faculty of Health Sciences, Max Rady College of Medicine, University of Manitoba, Winnipeg, MB Canada

**Keywords:** Cell death in the nervous system, Stroke

## Abstract

Ischemic stroke is a common disease with high morbidity and mortality. Remote ischemic preconditioning (RIPC) can stimulate endogenous protection mechanisms by inducing ischemic tolerance to reduce subsequent damage caused by severe or fatal ischemia to non-ischemic organs. This study was designed to assess the therapeutic properties of RIPC in ischemic stroke and to elucidate their underlying mechanisms. Neurobehavioral function was evaluated with the modified neurological severity score (mNSS) test and gait analysis. PET/CT was used to detect the ischemic volume and level of glucose metabolism. The protein levels of cytochrome c oxidase-IV (COX-IV) and heat shock protein 60 (HSP60) were tested by Western blotting. TUNEL and immunofluorescence staining were used to analyze apoptosis and to observe the nuclear translocation and colocalization of apoptosis-inducing factor (AIF) and endonuclease G (EndoG) in apoptotic cells. Transmission electron microscopy (TEM) was used to detect mitochondrial-derived vesicle (MDV) production and to assess mitochondrial ultrastructure. The experimental results showed that RIPC exerted significant neuroprotective effects, as indicated by improvements in neurological dysfunction, reductions in ischemic volume, increases in glucose metabolism, inhibition of apoptosis, decreased nuclear translocation of AIF and EndoG from mitochondria and improved MDV formation. In conclusion, RIPC alleviates ischemia/reperfusion injury after ischemic stroke by inhibiting apoptosis via the endogenous mitochondrial pathway.

## Introduction

Ischemic stroke is caused by emboli or vascular disease and leads to ischemia and hypoxia in brain tissue^1^. It is a major cause of death and long-term disability in adults worldwide. As the population ages, the stroke burden is expected to increase substantially; in addition, the prevalence of cerebrovascular disease-induced stroke is gradually increasing and imposes heavy social and economic burdens on individuals and families^2^. Although the restoration of cerebral blood flow is an important process, cerebral tissue that undergoes long-term ischemia and hypoxia experiences a certain degree of damage or dysfunction during blood flow reperfusion; this damage, called cerebral ischemia/reperfusion injury (CIRI), aggravates ischemic injury of the brain and is mainly characterized by cell necrosis and apoptosis^3^. Therefore, finding protective measures against CIRI and identifying methods for reducing CIRI have become the focuses of research on ischemic stroke.

Remote ischemic preconditioning (RIPC) is a temporary, gentle intervention below the threshold of injury that allows distant organs to achieve tolerance against subsequent prolonged ischemic episodes^4^. Studies have shown that animals that undergo brief limb ischemia exhibit smaller cerebral infarctions than those that do not undergo this intervention before ischemia^5–7^ and that RIPC can alter peripheral immune responses, alleviate brain edema and reduce apoptosis and necrosis of nerve cells to counteract later cerebral ischemic events^8,9^. However, the overall results of a study investigating RIPC in patients with acute ischemic stroke (AIS) treated with intravenous thrombolysis were neutral^10^. Some methodological restrictions may have been related to these results, but the low recanalization rate (20–30%) in this patient population may have been another important factor^11^. Even so, RIPC has been corroborated to be well tolerated in patients with AIS, and it may benefit these patients by reducing the risk of cerebral infarction, increasing cerebral tolerance to ischemic injury, improving cerebral perfusion status and exerting other beneficial effects^10,12,13^. Animal experiments and clinical trials on RIPC have both provided substantial evidence regarding its use in the treatment of ischemic stroke, but the specific mechanisms underlying the effects of RIPC remain elusive.

Modern medical research has revealed that the pathophysiological mechanism of ischemic stroke is related to reactive oxygen species (ROS) generation, mitochondrial damage, intracellular calcium overload, apoptosis, immune inflammatory injury, and other factors^14,15^. Mitochondria are the powerhouses of the cell, consuming oxygen to produce sufficient amounts of energy for the maintenance of normal cellular processes and playing vital roles in the development, proliferation, differentiation and dendritic remodeling of the nervous system^16^. As the most active organ in terms of energy metabolism, the brain has extremely high energy requirements. Impairment of the structure and function of mitochondria may induce degradation of damaged mitochondria by proteasomes or through mitophagy, reducing energy production and accelerating apoptosis^17–20^. Therefore, the removal of damaged mitochondrial parts is essential for restoration of mitochondrial function. To maintain mitochondrial homeostasis and effective functioning, cells and mitochondria have developed a wide range of mitochondrial quality control mechanisms to repair damaged mitochondria, such as mitochondrial biogenesis, fission/fusion, mitophagy and mitochondrial-derived vesicle (MDV) formation^21,22^. MDV formation is an emerging mitochondrial quality control pathway; MDVs selectively induce damaged mitochondrial proteins and lipids to form vesicles for degradation in peroxisomes or lysosomes to maintain mitochondrial integrity and reduce apoptosis^23,24^. We hypothesized that RIPC administered before the onset of ischemic stroke would improve mitochondrial structure. In this experiment, we observed MDV budding events and the translocation of apoptosis-inducing factor (AIF) and endonuclease G (EndoG) from mitochondria to nuclei and investigated whether the mitochondrial pathway is involved in the protective mechanism of RIPC against CIRI.

## Materials and methods

### Animals

Healthy adult C57BL/6 mice weighing 23–26 g and aged 8–9 weeks were provided by the SPF Laboratory Animal Center of Southwest Medical University. All of the animals were housed in the same animal care facility under standard temperature (23 ± 2 °C), lighting (12-h light/dark cycle) and relative humidity (65 ± 5%) conditions and with free access to food and water. The experimental protocol complied with the guidelines of the People’s Republic of China on experimental animals. The animal protocol was approved by the Animal Ethical Committee of the Animal Center of Southwest Medical University (Luzhou, Sichuan), and the experimental procedures were optimized to minimize the number of animal deaths and reduce the pain felt by the experimental animals. The mice were randomly divided into four groups: the sham operation (sham) group, the cerebral ischemia/reperfusion (I/R) group, the RIPC group, and the RIPC + I/R group.

### RIPC

Mice were anesthetized with 1% pentobarbital sodium. A 1-cm-long incision was made at the base of the thigh. Aneurysm clips were used to occlude the bilateral femoral arteries for 3 min and were then loosened for 5 min of reperfusion; this cycle was repeated three times. Occlusion of femoral artery blood flow in the mice was characterized by cyanosis, limb swelling, loss of the dorsal foot pulse, and decreased skin temperature. The RIPC + I/R mice underwent RIPC for 48 h before cerebral I/R.

### Cerebral I/R models

A model of middle cerebral artery (MCA) occlusion (MCAO) was generated as previously described^25^. Briefly, each mouse was weighed and anesthetized with 1% pentobarbital sodium via intraperitoneal administration. The right common carotid artery (CCA) was exposed, and the external carotid artery (ECA) was ligated to prevent backflow of blood. A 6–0 nylon suture with a 0.21-mm diameter and a silicone-coated tip (Doccol, USA) was introduced from the CCA through the internal carotid artery (ICA) to occlude the MCA under a stereoscopic microscope. Reperfusion was induced by withdrawal of the filament after 90 min of occlusion, after which the animal was returned to a quiet room at 25 ± 1 °C and fed for 48 h. For each sham-operated mouse, the CCA, ICA, and ECA were exposed, and the skin was then sutured without any treatment.

### Behavioral tests

A modified neurological severity score (mNSS) test was performed to evaluate neurological deficits at 48 h after cerebral I/R^26,27^. The neurological deficit scores integrated exercise, sensory, balance, and reflex scores (normal score, 0; highest score, 18). Severity was classified as follows: 1–6 points, mild injury; 7–12 points, moderate injury; and 13–18 points, severe injury. One point was awarded for failure to complete a specific task or for the presence of a reflex that disappeared; in other words, the higher the cumulative score, the more severe the neurological impairment in mice.

### Gait analysis

A TreadScan Gait Analysis System (CleverSys. Inc., Reston, VA, USA) that included a transparent treadmill belt and a high-speed camera was used to obtain and analyze the footprints and gait of the mice from each group at 48 h after cerebral I/R. The lights on the TreadScan^TM^ were turned on for 5 min before the test to reduce illumination fluctuation. The treadmill was composed of a transparent electric treadmill belt with an angled mirror mounted below. A high-speed digital camera was installed to record the ventral side of the treadmill strap reflected in the mirror, and a background image was collected before each mouse was placed on the treadmill. The running speed of each mouse on the treadmill was 6 cm/s, with a maximum captured frame count of 2000 and a frequency of 100 frames/s. The calibration and footprint data files were loaded or generated before analysis. Stance time, swing time, stride length, print area, foot pressure and running speed were measured in this study.

### PET/CT

A micro-positron emission tomography (PET)/computed tomography (CT) scanner (Siemens, Germany) was used to perform ^18^F-fluorodeoxyglucose (^18^F-FDG) PET and CT scans on each mouse. All mice were prevented from drinking water and fasted for at least 6 h before the PET/CT scan. ^18^F-FDG (50 µCi-100 µCi for each mouse) was injected via the tail vein after intraperitoneal anesthesia with 1% pentobarbital sodium. Micro-PET/CT imaging was performed for 40 min after ^18^F-FDG injection. Each mouse was attached to a stent, placed on a PET/CT scanning bed, and subjected to PET scanning immediately followed by CT scanning (with the brain as the center point). The micro-PET/CT images were used to assess the infarct site and the range of ^18^F-FDG uptake and to delineate the standardized uptake value (SUV).

### Histopathology

The mice were sacrificed at 2 days after I/R. The brain tissues of the mice were removed rapidly and sliced into 8-µm-thick coronal sections on a freezing microtome. The brain sections were stained with hematoxylin (Jiancheng Biotech) for 5 min and differentiated with 1% hydrochloric acid alcohol for 5 s. The sections were then placed in ammonia water for 10 s, stained with eosin for 3 min, dehydrated, cleared with alcohol and xylene, and finally sealed with neutral balsam. Histopathological changes in the ischemic brains were observed under a microscope.

### TUNEL analysis

The sections were rinsed 3 times for 5 min each with phosphate-buffered saline (PBS), permeabilized in proteinase K solution (20 μg/ml in 10 mM Tris/HCL, pH 7.6) at 37 °C for 15 min, rinsed with PBS for 3×5 min, and incubated in TUNEL reaction mixture (Roche) for 1 h at 37 °C in a humidified atmosphere in the dark. The TUNEL-positive nuclei were stained with fluorescein isothiocyanate (red), the samples were rinsed with PBS for 3×5 min, and all nuclei were stained with DAPI (blue). The photomicrographs were analyzed by fluorescence microscopy.

### Western blot analysis

Brain samples were collected from the different groups and homogenized in RIPA lysis buffer with protease inhibitors (Beyotime Biotech, China). The brain sample proteins were separated by 12% sodium dodecyl sulfate polyacrylamide gel electrophoresis and transferred to PVDF membranes (100 V, 70 min). The membranes were blocked with 5% nonfat dry milk in Tris-buffered saline with 0.05% Tween 20 (TBST) and incubated with rabbit anti-heat shock protein 60 (HSP60) (1:1000, Cell Signaling), mouse anti-cytochrome c oxidase-IV (COX IV) (1:1000, Cell Signaling) and mouse anti-glyceraldehyde-3-phosphate dehydrogenase (GAPDH) (1:10000, Abcam) primary antibodies at 4 °C overnight. After rinsing in TBST 3 times for 10 min, the membranes were incubated with horseradish peroxidase-conjugated goat anti-mouse/anti-rabbit IgG secondary antibodies (1:2000, Bio-Rad) at room temperature for 1 h and then rinsed with PBS for 3×10 min. The immunoblots were detected by enhanced chemiluminescence (ECL), and the protein levels were analyzed by Quantity One software.

### TEM

Transmission electron microscopy (TEM) was performed to observe MDV formation. Brain tissues were fixed in 2.5% glutaraldehyde (Sigma) in phosphate buffer overnight at 4 °C. Next, the samples were fixed in 1% osmium tetroxide for 2 h, dehydrated with a graded series of acetone washes, infiltrated with propylene epoxide, and embedded in Epon 618. After sample preparation, 90–100-nm-thick sections were mounted onto a 200-mesh copper grid and imaged with a JEM-1400 series 120 kV transmission electron microscope (JEOL, Japan) with an integrated high-sensitivity complementary metal oxide semiconductor (CMOS) camera.

### Immunofluorescence

Brain slices were washed with PBS for 15 min, permeabilized in 0.3% Triton X-100 solution for 10 min at room temperature, and then blocked in 1% bovine serum albumin (BSA, Beyotime, China) for 1 h. The primary antibodies used in this study included rabbit anti-AIF and rabbit anti-EndoG (Abcam, 1:50), which were diluted in PBS containing 1% BSA. An Alexa Fluor 488-conjugated goat anti-rabbit (1:500) secondary antibody was purchased from Invitrogen. Primary antibody incubation was performed at 4 °C overnight, and secondary antibody incubation was performed at room temperature for 1.5 h. Finally, the slices were covered with fluorescence mounting medium (Dako) on glass slides.

### Statistical analysis

All data in this study are presented as the mean ± standard error of the mean (SEM) and were analyzed using GraphPad Prism 6 software. Statistical differences between multiple groups were analyzed using one-way ANOVA, and *P* < *0.05* was considered to indicate statistical significance.

## Results

### Histopathological structure of brain tissue

**Figure 1:**
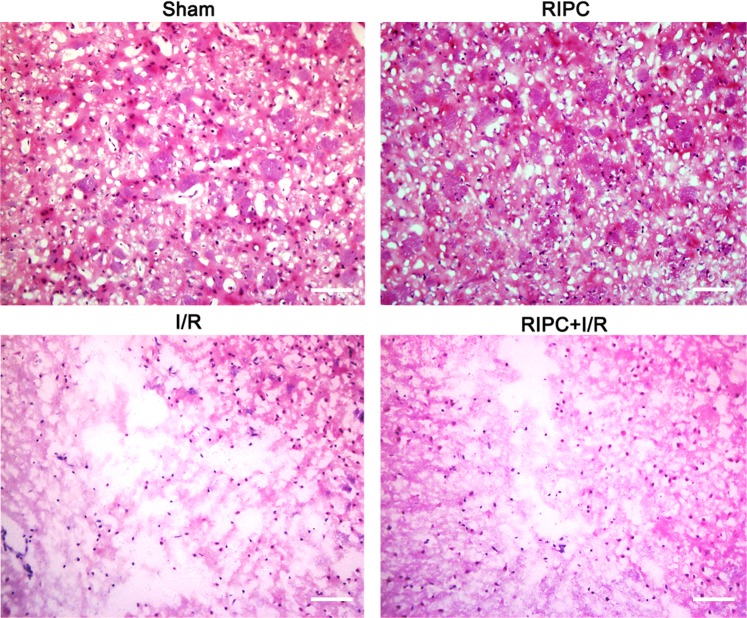
Histopathological structure of the brain tissue in the different groups. The brain tissue around the ischemic area in the RIPC + I/R group was more intact and compact than that in the I/R group. Bar = 100 μm.

Hematoxylin-eosin (H&E) staining showed normal tissue structure in the sham and RIPC groups. There were a series of morphological abnormalities, such as loose tissue and sparse, swollen cells, in the brain tissue after CIRI. The brain tissue around the ischemic area in the RIPC + I/R group was more intact and compact than that in the I/R group (Fig. 1).

### RIPC attenuated neurological impairments after CIRI

**Figure 2:**
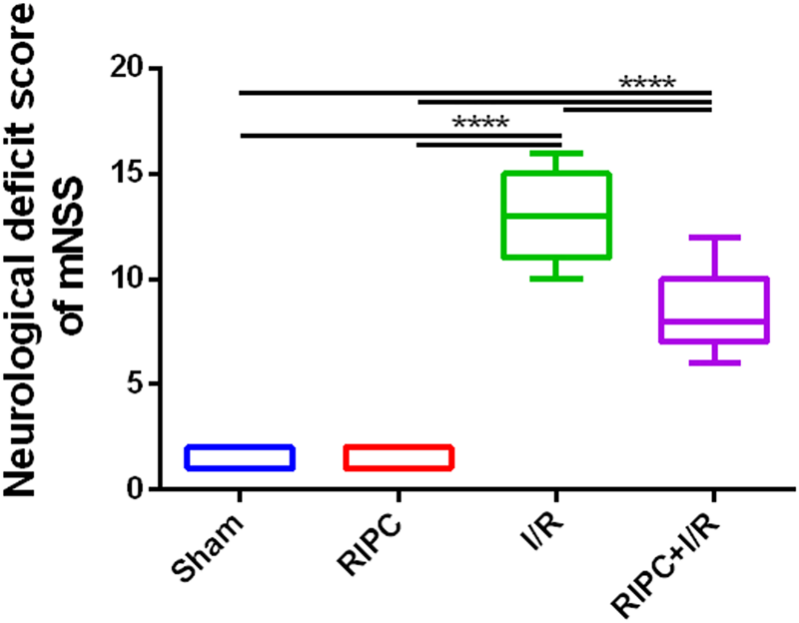
Behavioral evaluation by the mNSS test. Compared with that of the I/R group, the score of the RIPC + I/R group was significantly reduced. ****P < 0.0001.

The mNSS test was used to evaluate the neuroprotective effects of RIPC at 48 h after CIRI. As shown in Fig. 2, mice with ischemic stroke exhibited obvious neurological dysfunction. The neurological deficit score of the RIPC + I/R group was significantly lower than that of the I/R group. However, no neurological deficit was detected in the sham and RIPC groups.

### Effect of RIPC on gait in mice with ischemic stroke

**Figure 3:**
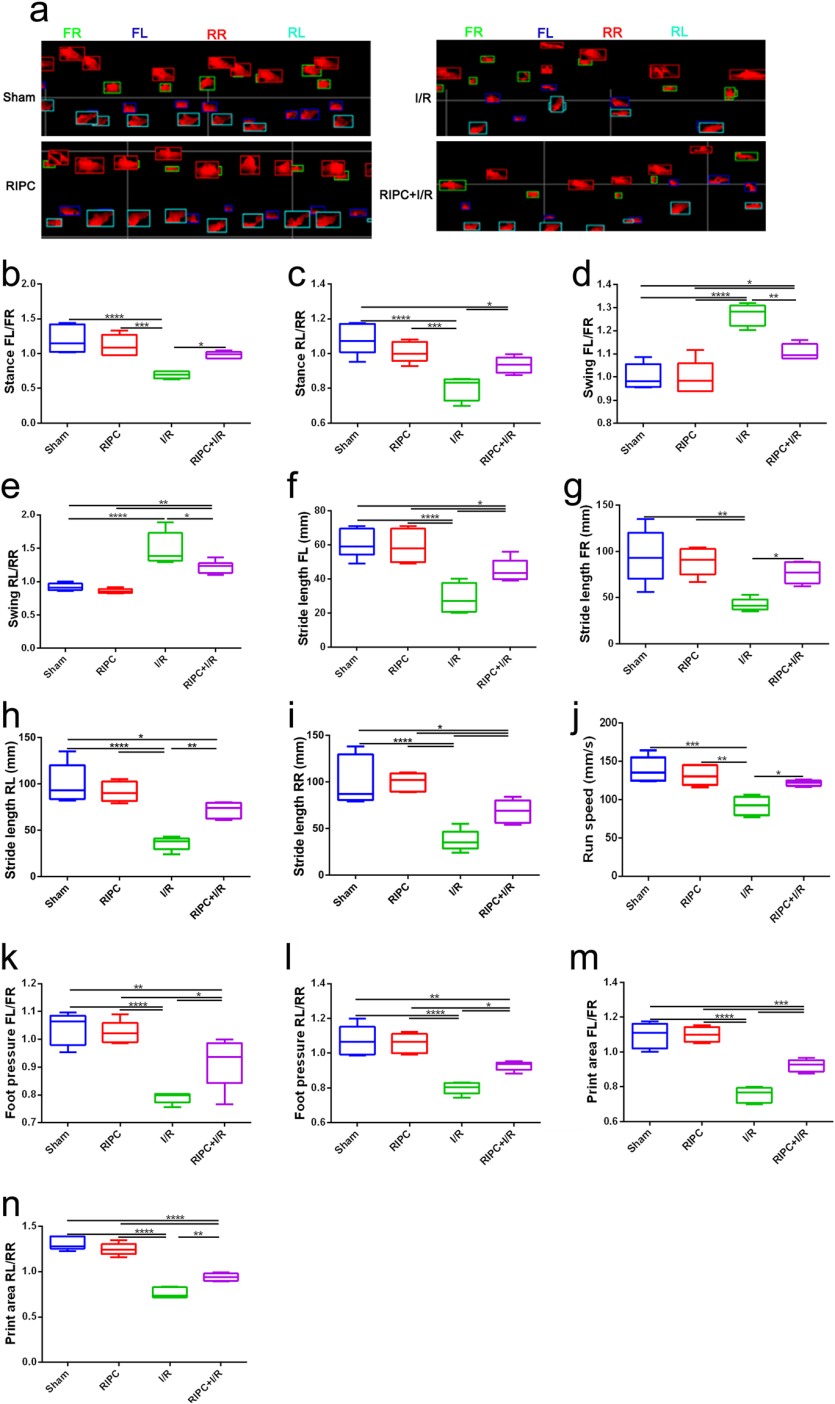
Changes in gait parameters in the different groups. (**a**) Representative footprints of the rats in the different groups obtained by the analysis software. Green, FR (front right foot); dark blue, FL (front left foot); red, RR (rear right foot); sky blue, RL (rear left foot). (**b–p**) The data show significant differences in stance time, swing time, stride length, running speed, foot pressure, print area and stride number among the 4 groups. *P < 0.05, **P < 0.01, ***P < 0.001, ****P < 0.0001.

The TreadScan^TM^ system was used to analyze whether RIPC treatment resulted in changes in neurological function with regard to specific gait parameters during forced locomotion on a treadmill. The data showed that RIPC could improve the run speed and stride lengths of all four paws in ischemic mice. The stance, foot pressure, and print area in the RIPC + I/R group were significantly higher than those in the I/R group; however, the swing in the RIPC + I/R group was obviously lower than that in the I/R group. These results indicate that RIPC treatment affects gait patterns in ischemic stroke mice (Fig. 3).

### RIPC reduced infarct size and increased glucose metabolism

**Figure 4:**
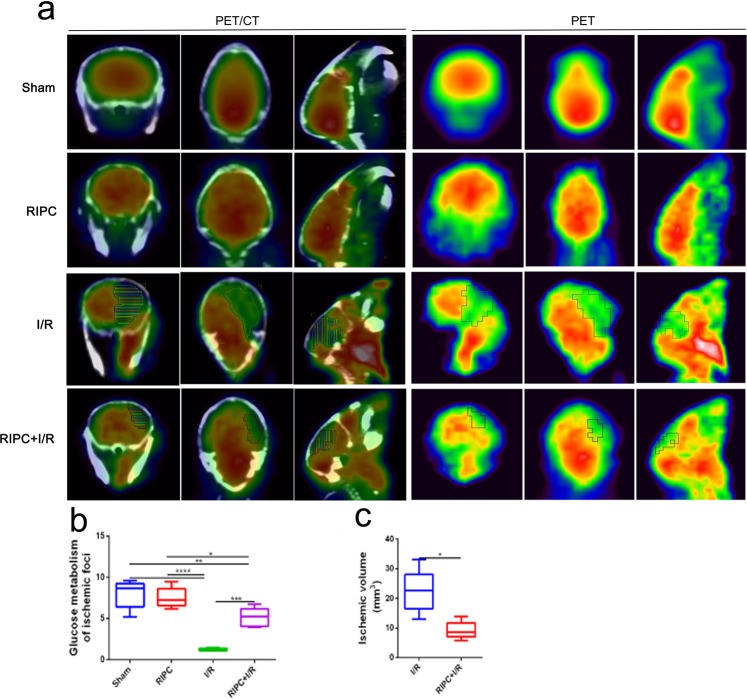
Results of ^18^F-FDG micro-PET/CT scans in the different groups. (**a**) Representative coregistered PET/CT (left) and PET (right) scan images, including axial, coronal and sagittal (R) images, of the mouse brains. (**b**) Quantitative analysis of glucose metabolism in ischemic foci. (**c**) Infarct volumes in the I/R and RIPC + I/R groups. *P < 0.05, **P < *0.01*, ***P < *0.001*, ****P < *0.0001*.

^18^F-FDG micro-PET/CT scans were used to assess the infarct volume and glucose metabolism in the ischemic foci at 48 h after CIRI. There were no ischemic foci in the sham and RIPC groups, and the glucose metabolism levels in these groups were significantly higher than those in the I/R and RIPC + I/R groups. The results also showed that the ischemic volume in the RIPC + I/R group was significantly smaller than that in the I/R group. Compared with that in the I/R group, the level of glucose metabolism was increased significantly in the RIPC + I/R group (Fig. 4).

### RIPC suppressed apoptosis

**Figure 5:**
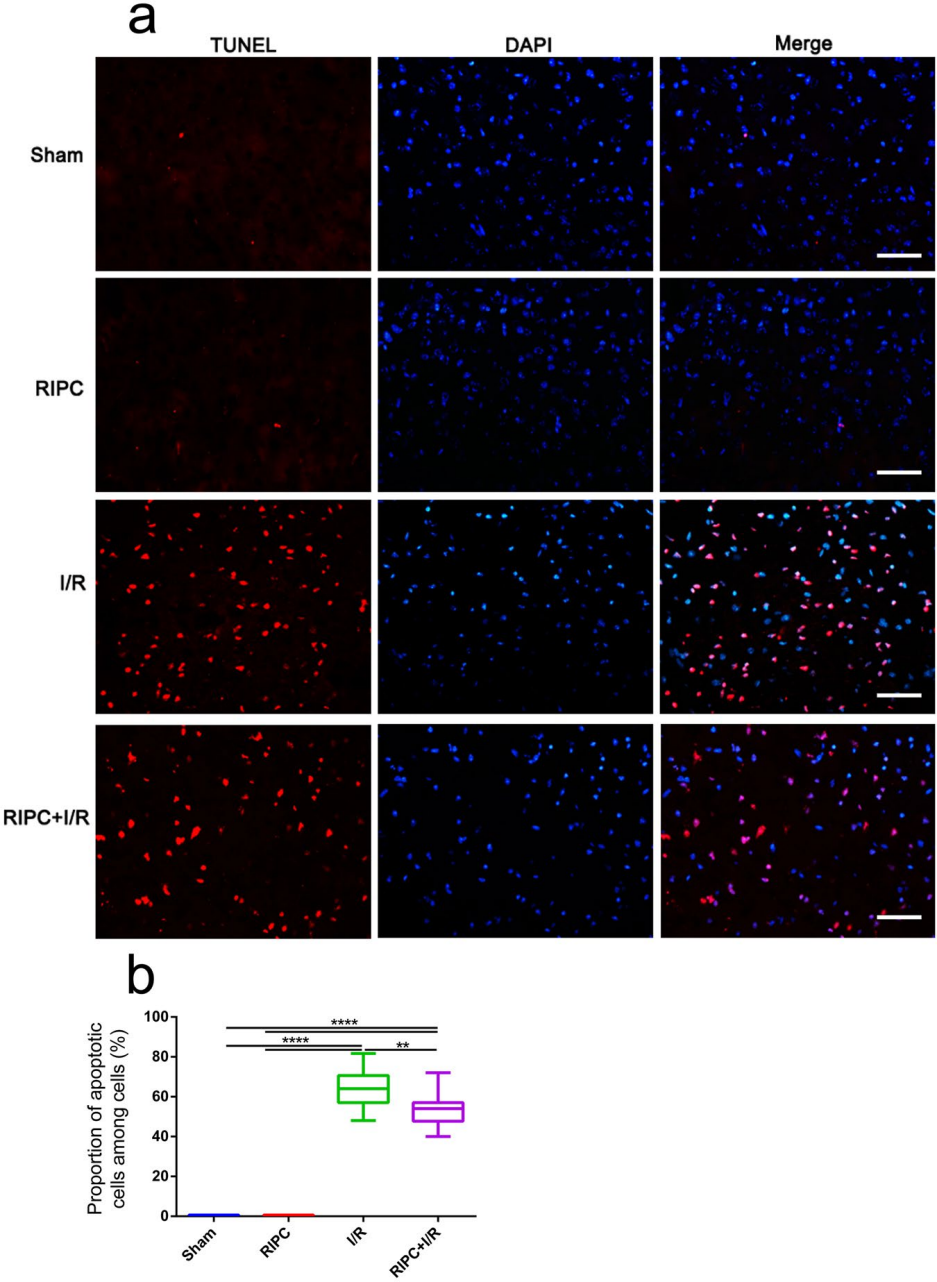
Apoptotic cells were analyzed by TUNEL staining. (**a**) Apoptotic cells were labeled with fluorescein isothiocyanate (red), and all nuclei were stained with DAPI (blue). (**b**) Percentage of apoptotic cells relative to total cells. **P < 0.01, ****P < 0.0001. Bar = 50 μm.

Apoptotic cells were detected by TUNEL staining, and the proportion of apoptotic cells in the RIPC + I/R group was significantly lower than that in the I/R group. In addition, only a few apoptotic cells were observed in the sham and RIPC groups (Fig. 5).

### Expression of COX IV and HSP60 proteins

**Figure 6:**
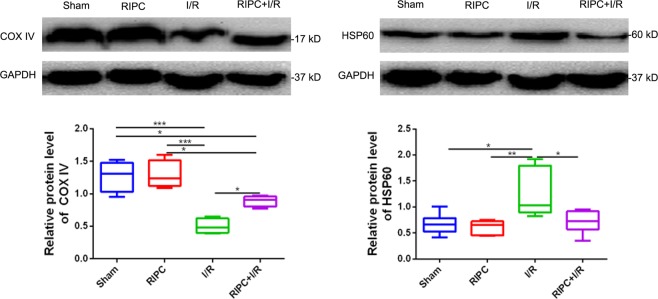
The protein expression levels of COX IV and HSP60 were examined by Western blotting. *P < 0.05, **P < 0.01, ***P < *0.001*.

Figure 6 shows that COX IV protein levels were sharply reduced after cerebral ischemia and that the COX IV protein level in the RIPC + I/R group was significantly higher than that in the I/R group. HSP60 protein expression in the I/R and RIPC + I/R groups was significantly higher than that in the sham and RIPC groups. Moreover, the HSP60 protein level in the RIPC + I/R group was significantly lower than that in the I/R group.

### AIF and EndoG translocation and colocalization in apoptotic cells

**Figure 7:**
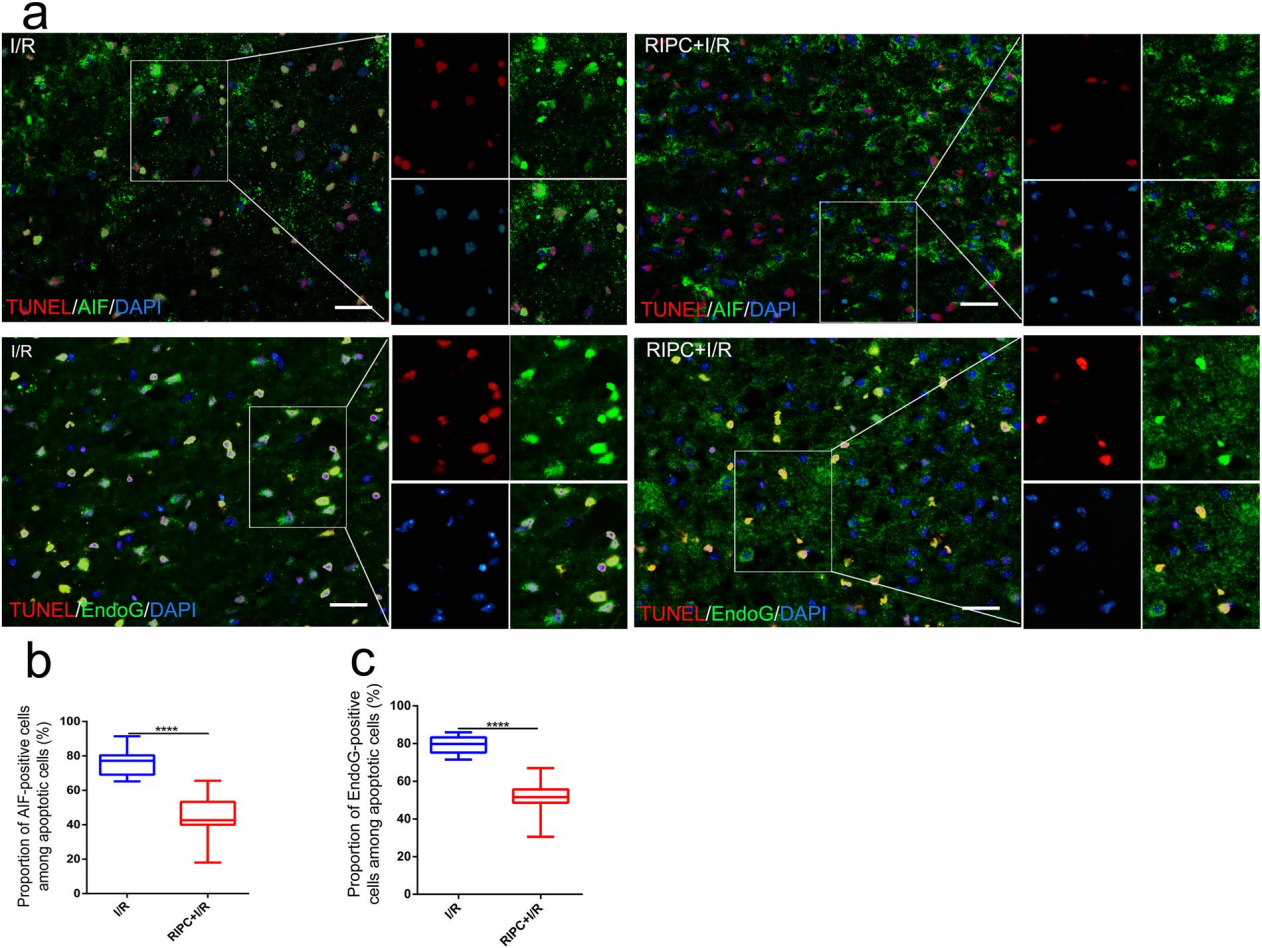
The colocalization of AIF and EndoG proteins in apoptotic cells was determined by TUNEL and immunofluorescence staining. (**a**) Immunofluorescence staining for AIF (green), EndoG (green) and DAPI (blue) and staining of apoptotic cells (red). (**b**,**c**) Proportions of TUNEL-positive cells exhibiting colocalization of AIF and EndoG that translocated from mitochondria to nuclei relative to the total TUNEL-positive cells in the different groups. ****P < *0.0001*. Bar = 50 μm.

As shown in Fig. 7, translocation of AIF and EndoG from mitochondria to nuclei and colocalization of these proteins in apoptotic cells were observed in the ischemic penumbra after CIRI. The proportion of TUNEL-positive cells that exhibited colocalization of AIF and EndoG that translocated from mitochondria to nuclei relative to the total TUNEL-positive cells in the RIPC + I/R group was significantly lower than that in the I/R group (Fig. 7).

### MDV formation

**Figure 8:**
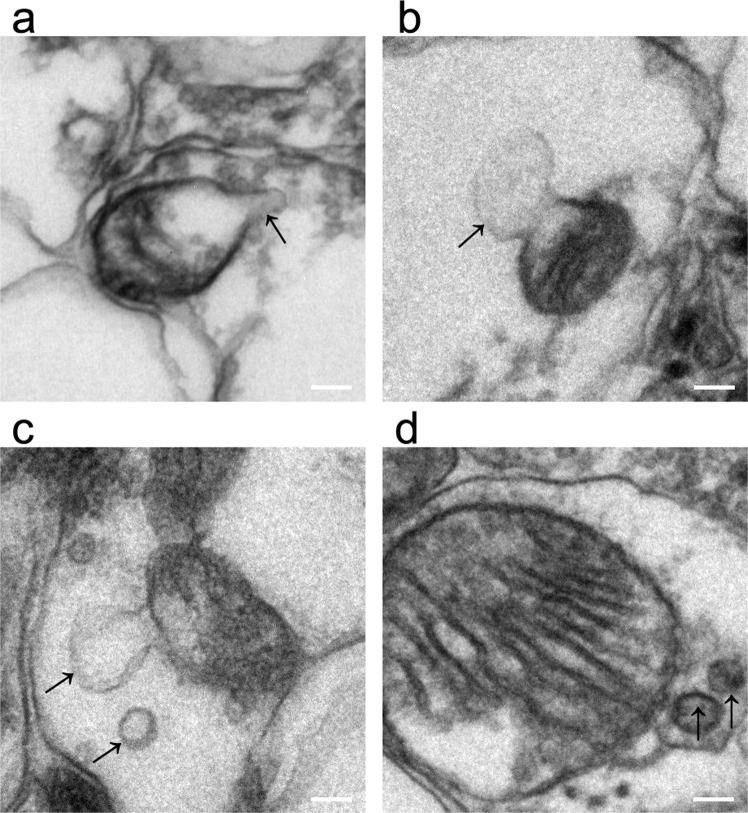
Formation of MDVs. The entire process of MDV formation (arrows) was observed from germination to exfoliation (**a–d**). Bar = 100 nm.

MDV formation, specifically budding events, were visualized using TEM. Importantly, vesicular structures between 50 and 200 nm in diameter were identifiable on the surfaces of mitochondria (Fig. 8). In this study, we observed that the membranes of these vesicles were clearly adjacent to the mitochondrial membranes.

### RIPC promoted MDV budding after CIRI

**Figure 9:**
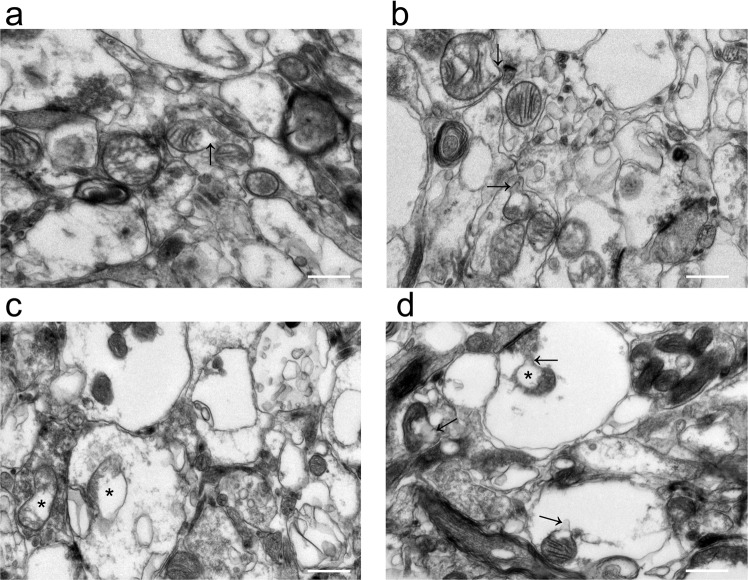
Identification of mitochondrial budding events by TEM. Mitochondrial buds are marked with arrows, and mitochondrial vacuoles are marked with asterisks (**a–d** represent the sham, RIPC, I/R and RIPC + I/R groups, respectively). Bar = 500 nm.

To further verify whether I/R induces ultrastructural changes in mitochondria in the brain, we next performed experiments in mouse models of ischemic stroke. As shown in Fig. 9, in the sham and RIPC groups, mitochondrial cristae were clear, almost no mitochondrial vacuolization occurred, and a few MDV germination events occurred. In the I/R group, mitochondria showed swelling, disorder of sparse cristae and fracture; numerous mitochondrial vacuoles were observed; and few MDV germination events occurred. However, the mitochondria in the RIPC + I/R group were slightly swollen with less vacuolation than those in the I/R group, the mitochondrial cristae were vague but still visible, and more MDV budding events occurred than in the other groups.

## Discussion

In this study, we observed that RIPC significantly reduced the proportions of TUNEL-positive cells that exhibited colocalization of AIF and EndoG proteins that were translocated from mitochondria to nuclei relative to the total TUNEL-positive cells and that RIPC inhibited apoptosis in the ischemic penumbra after CIRI. The above results may have been correlated with the findings regarding mNSS scores, gait, infarct volume, glucose metabolism and COX IV and HSP60 protein levels. We further found that mitochondria in the RIPC + I/R group were slightly swollen with less vacuolation and more MDV budding events than those in the I/R group. These data suggest that RIPC alleviates CIRI by inhibiting apoptosis associated with ischemic stroke via an endogenous mitochondrial pathway related to mitochondrial quality control.

Remote ischemic conditioning (RIC) is an attractive phenomenon whereby intermittent ischemia and reperfusion cycles applied to a single vascular bed, tissue, or organ endow global protection, rendering distal tissues and organs resistant to I/R injury (IRI)^28^. Previous studies have shown that RIC can reduce myocardial infarct size in patients with ST-elevation myocardial infarction treated by thrombolysis and that it can increase myocardial salvage in patients with acute myocardial infarction^29,30^. Accumulating evidence has revealed that RIC also plays a protective role in brains subjected to ischemia^31–36^. RIC can be divided into three types according to the onset time: (1) RIPC, in which RIC starts before the onset of brain ischemia; (2) RIPerC, in which RIC starts after the onset of ischemia and before reperfusion of the brain; and (3) RIPostC, in which RIC starts during the reperfusion period^12^. RIPC can reduce recurrent strokes and improve cerebral perfusion in patients with intracranial arterial stenosis^13^. Similar to RIPC, RIPerC and RIPostC have also been found to be effective in ameliorating ischemic stroke; in fact, RIPerC is superior to RIPC in reducing brain infarct volume^37,38^. Prevention is better than cure for disease, so exploration of the effects of RIPC is of great significance.

Two therapeutic time windows for RIPC-mediated protection against ischemic stroke have been identified in past studies; immediate protection from preconditioning is possible after just 30–60 min, and persistent protection is possible after 24 to 72 h^39–41^. The former is called rapid ischemic tolerance, and the latter is called delayed ischemic tolerance. This study was a proof of concept study, and we chose delayed RIPC because its protective effect on the brain peaks at 48 h^42^.

Ischemic stroke can cause severe neurological impairment, such as hemiplegia and hemidysesthesia. In this experiment, we found that RIPC decreased mNSS scores in mice after ischemic stroke. TreadScan^TM^ analysis has been shown to be a simple, sensitive, accurate, and objective method of detecting gait changes in rodent models, such as models of stroke and Parkinson’s disease^43,44^. In this study, we found that the stance, running speed, print area and foot pressure of the RIPC + I/R group were significantly higher than those of the I/R group; in addition, the stride length (all four paws) of the RIPC + I/R group was significantly longer than that of the I/R group, but the swing time of the RIPC + I/R group was lower than that of the I/R group. These results suggest that RIPC can reduce neurological deficits and promote neural function recovery in mice with ischemic stroke.

The brain is a very energy-consuming organ that performs its normal function through aerobic metabolism. Glucose is an essential metabolic substrate for energy production, ribonucleotide biosynthesis, and redox balance maintenance. Alterations in glucose metabolism are considered to be important pathological mechanisms of ischemic stroke^45–47^. In addition to being involved in metabolic energy production, glucose is also involved in the production and elimination of ROS, which cause oxidative damage to membrane lipids, proteins and nucleic acids^48^. Støttrup found that ischemic preconditioning (IPC) inhibits glycolysis and increases glucose oxidation during reperfusion by tracing glucose metabolism in isolated perfused rat hearts exposed to 40 min of global no-flow ischemia^49^. In this study, ^18^F-FDG micro-PET/CT scans demonstrated that the volume of the ischemic area in the RIPC + I/R group was smaller than that in the I/R group. Moreover, the higher level of glucose metabolism in the RIPC + I/R group than in the I/R group is noteworthy, as it implies that increased cerebral glucose uptake due to RIPC may be related to cell survival and proliferation or to reduced cell death. However, the specific mechanism still needs to be further studied.

Mitochondria are the energy factories of cells, and their structural and functional integrity is the basis of energy metabolism. Recent studies have shown that plasma or plasma dialysate from pigs undergoing RIPC not only reduces infarct size and mitochondrial ROS production but also improves ADP-stimulated complex I respiration, ATP production and calcium retention capacity in isolated perfused rat hearts subjected to 30 min of global ischemia followed by 120 min of reperfusion; in addition, these studies have shown that the plasma dialysate increases the viability of mouse cardiomyocytes after hypoxia/reoxygenation^50,51^. A clinical study has demonstrated that RIPC also improves mitochondrial and contractile function in right atrial tissue in patients with double- or triple-vessel coronary artery disease^52^. Therefore, mitochondria are fundamental targets for cardioprotective strategies, and conservation of mitochondrial function is the focus of treatments to reduce IRI^53^. After restoration of blood flow, oxidative stress is activated, which results in abnormal mitochondrial structure and function followed by sharp reductions in energy supply and cell death, which are important mechanisms of CIRI^54^. COX is a rate-limiting enzyme in the respiratory electron transport chain in the mitochondrial inner membrane and takes center stage in metabolic control, cell signaling and survival^55^. Its IV subunit guides COX assembly, maintains cell structure stability and is a mitochondrial biogenetic marker and key enzyme for the regulation of oxidative phosphorylation and cell productivity^56^. Suppression of COX IV expression not only reduces cytochrome c oxidase-dependent respiration, total respiration, and ATP levels but also sensitizes cells to apoptosis^57^. HSP60 is a mitochondrial chaperonin protein that assists in the proper folding, assembly and translocation of proteins and peptides and is often used as a reliable indicator of mitochondrial stress intensity and damage^58,59^. Upregulation of HSP60 is another response that occurs after exposure to many stressors and is indicative of mitochondrial biogenesis^60^. In this study, the protein level of COX IV was significantly higher in the RIPC + I/R group than in the I/R group, but the expression of HSP60 was obviously lower in the RIPC + I/R group. However, severe mitochondrial swelling, sparse cristae and vacuolization were observed after cerebral ischemia, indicating that mitochondrial structure and function were destroyed. The mitochondria in the RIPC + I/R group were mildly swollen, the cristae were blurred by visible, and mitochondrial vacuolization was reduced, indicating that RIPC reduced the degree of mitochondrial swelling and vacuolization, improved mitochondrial structure and affected mitochondrial biogenesis. However, this study did not further analyze mitochondrial function in terms of mitochondrial respiration or mitochondrial ATP generation, which is a limitation. Increases in mitochondrial mass obviously improve the overall oxidative functions and energy states of brains with IRI. This may be an endogenous neuroprotective response against CIRI.

Studies have demonstrated that the ischemic core is rapidly infarcted after cerebral ischemia but that the ischemic penumbra can be saved within a certain time period^61^. Moreover, the cell death caused by CIRI is mainly delayed neuronal death that occurs via apoptosis in the ischemic penumbra^62–65^. Apoptosis is an active cell death process that is regulated by genes and is mainly divided into the mitochondrial pathway, death receptor pathway, and endoplasmic reticulum pathway^66^. Mitochondria are the main sites of oxidative respiration in cells and are the regulatory centers of the mitochondrial pathway. When mitochondria are damaged and irreversible loss of the membrane potential occurs, cell death is inevitable^67–69^. After mitochondrial injury, AIF and EndoG are released from the mitochondria into the cytoplasm and subsequently translocate to the nucleus, which is a necessary step for stimulation of apoptosis^70,71^. In the present study, we observed that large amounts of AIF and EndoG were released from mitochondria, translocated to nuclei and localized in apoptotic cells in ischemic mice. However, significantly less AIF and EndoG translocated from mitochondria to nuclei in the RIPC + I/R group than in the I/R group. These data demonstrate that RIPC inhibited the release and translocation of AIF and EndoG and reduced apoptosis mediated by the endogenous mitochondrial pathway; however, the exact protective mechanism is still unclear.

To defend mitochondria against damage, cells and mitochondria have developed extensive mitochondrial quality control mechanisms to remove damaged mitochondrial cargo and ensure that mitochondria maintain their normal functions^72^. MDV formation is one of these defense mechanisms; MDVs transport damaged mitochondrial cargo to lysosomes for degradation to maintain mitochondrial integrity^73^. MDVs, which are single-membrane structures derived from the outer membranes of mitochondria or double-membrane structures including the mitochondrial inner and outer membranes and a mitochondrial matrix^74^, represent the first line of defense for removal of damaged cargo before further damage is done to the organelles. In this experiment, we found that numerous damaged mitochondria emerged after cerebral ischemia; at the same time, few MDV budding events were observed. However, RIPC significantly increased MDV budding in ischemic mice and restored mitochondrial structure. These findings indicate that RIPC may be beneficial for the budding and formation of MDVs and thus for the maintenance of mitochondrial structure and further reductions in apoptosis.

### Summary

In conclusion, RIPC can effectively improve the symptoms of neurological deficits and reduce the volume of ischemic lesions after cerebral ischemia. We speculate that RIPC increases the resistance of cells to ischemic insults by promoting MDV formation to remove damaged segments of mitochondria, maintain mitochondrial structural and functional integrity, decrease mitochondrial degradation and decrease nuclear translocation of AIF and EndoG from mitochondria to inhibit the mitochondrial pathway. These changes consequently reduce apoptosis to achieve neuroprotection. Further studies and an optimized experimental design taking the results of this study into consideration are required to better understand and verify the mechanisms.

## Data Availability

The datasets generated during and/or analyzed during the current study are available from the corresponding author on reasonable request.
